# Effects of Monacolin K in Nondiabetic Patients with NAFLD: A Pilot Study

**DOI:** 10.3390/nu15081887

**Published:** 2023-04-14

**Authors:** Andrea Da Porto, Debora Donnini, Fabio Vanin, Arianna Romanin, Martina Antonello, Paolo Toritto, Eleonora Varisco, Gabriele Brosolo, Cristiana Catena, Leonardo A. Sechi, Giorgio Soardo

**Affiliations:** 1 Clinica Medica, Department of Medicine, University of Udine, 33100 Udine, Italy; 2 Diabetes and Metabolism Unit, Department of Medicine, University of Udine, 33100 Udine, Italy; 3 Liver Unit, Department of Medicine, University of Udine, 33100 Udine, Italy; 4 Italian Liver Foundation, Area Science Park, Basovizza, 34149 Trieste, Italy

**Keywords:** bioimpedance, glutathione, liver ultrasound, liver steatosis, liver fibrosis, malondialdehyde

## Abstract

Non-alcoholic fatty liver disease (NAFLD) is a common chronic liver condition with significant risk of progression to steatohepatitis and cirrhosis. Therapeutic strategies in NAFLD include lifestyle changes mainly related to dietary interventions and use of drugs or nutritional components that could improve plasma lipid profiles and insulin sensitivity and decrease the local inflammatory response. In this study, we tested the effects of monacolin K, an inhibitor of HMCoA reductase. In a prospective, uncontrolled, open study, we treated 24 patients with NAFLD and mild hypercholesterolemia with 10 mg/day of monacolin K. At baseline and after 26 weeks, we measured in plasma liver tests, lipids, malondialdehyde, and oxidized glutathione, and assessed biochemical steatosis scores, liver elastography, and body composition with bioimpedance analysis. Monacolin K significantly reduced plasma alanine aminotransferase, cholesterol, triglycerides and the homeostatic model assessment (HOMA) index that indicated improved insulin sensitivity. No significant changes were found in body fat mass and visceral fat, nor in liver elastography, while the fatty liver index (FLI) was significantly decreased. Plasma levels of both malondialdehyde and oxidized glutathione were markedly reduced by monacolin K treatment, suggesting a reduction in oxidative stress and lipid peroxidation. In summary, this pilot study suggests possible benefits of monacolin K use in NAFLD patients that could be linked to a reduction in oxidative stress. This hypothesis should be further investigated in future studies.

## 1. Introduction

Non-alcoholic fatty liver disease (NAFLD) is a chronic hepatic condition characterized by accumulation of fat in hepatocytes and is diagnosed on liver biopsy when more than 5% of hepatocytes are repleted with fat in teetotalers or presence of low alcohol intake (less than 20 g/day of anhydrous ethanol for women and 30 g/day for men) [1]. NAFLD is the most common liver disorder in western countries with a growing prevalence [2] that could reach up to one third of the general population [3]. NAFLD is commonly considered the distinctive feature of the liver involvement in the metabolic syndrome [4] that is defined as the cluster of any three of the following five conditions: increased waist circumference, impaired fasting glucose (IFG), hypertriglyceridemia, low high-density lipoprotein cholesterol, and high blood pressure [5]. Insulin resistance is widely recognized as the underlying mechanism linking components of the metabolic syndrome and might play a major role also on NAFLD occurrence [4,5]. Metabolic studies demonstrated an association of central obesity with insulin-related metabolic responses in hepatocytes, leading to increased hepatic gluconeogenesis and very low-density lipoprotein production, and ultimately causing uncontrolled triacylglycerol accumulation [6].

Approximately one-third of NAFLD cases progress to nonalcoholic steatohepatitis (NASH), and approximately 20% of NASH progress to cirrhosis and, eventually, to hepatocellular carcinoma [3]. Insulin resistance, low-grade inflammation and oxidative stress with reduced antioxidant defense are well established features involved in the progression of liver damage in NAFLD [7,8]. Increased oxidative stress resulting from excess free radical generation impairs mitochondrial beta-oxidation of fatty acids [9] and promotes lipid peroxidation, thereby inducing chronic inflammation with progression of hepatocellular damage and liver fibrosis [10]. 

Nutraceuticals are biologically active compounds that are commonly used with the aim to improve health status, prevent diseases and delay senile decay. Because of their nutrition and therapeutic potentials, nutraceuticals are currently gaining substantial attention and are tested in many metabolic-related pathologic conditions including NAFLD [11]. Monacolin K, a metabolite of Monascus sp. (red yeast), is a potent inhibitor of 3-hydroxy-3-methylglutaryl coenzymeA (HMG-CoA) reductase and its consumption decreases circulating cholesterol and triglyceride levels [12]. For these reasons, monacolin K is frequently used as an alternative treatment in patients with hypercholesterolemia who are intolerant to statins or decide not to take statins. Early animal studies suggested that administration of monacolin K might be beneficial in experimental models of NAFLD by improving insulin sensitivity and decreasing obesity-related inflammation [13], but human studies are lacking. In this study, we examined the effects of chronic administration of monacolin K to nondiabetic patients with NAFLD and mild elevation of plasma cholesterol levels, and hypothesized a possible involvement of oxidative stress that was assessed by measurement of plasma markers.

## 2. Materials and Methods

### 2.1. Study Design, Setting, and Patients

In this prospective, observational study, we enrolled previously untreated patients with NAFLD who were consecutively referred to the outpatient clinic of the University Hospital of the University of Udine, Italy. Patients are referred to the clinic by general practitioners for assessment of changes in liver tests and/or plasma lipids. Predefined inclusion criteria were age > 18 years, diagnosis of NAFLD as obtained by ultrasound imaging with assessment of liver stiffness, and plasma cholesterol concentration > 200 mg/dL. Predefined exclusion criteria were: consumption of alcoholic beverages (any type); body mass index (BMI) > 35 kg/m^2^; pregnancy or use of estrogens; diabetes mellitus; any type of liver diseases other than NAFLD; glomerular filtration rate < 30 mL/min/1.73 m^2^; cognitive disorders; previous treatment with statins or other hypolipemic agents; previous coronary artery, cerebrovascular and peripheral vascular events; history of recent acute illness or cancer; contraindications to bioimpedance analysis (BIA). Before inclusion in the study and for at least one year, all NAFLD patients were recommended to eat a Mediterranean diet [2,14] and to refrain from consumption of alcoholic drinks. The same dietary recommendations were maintained throughout the intervention study. All patients were treated with monacolin K (Erba Vita, Chiesanuova, Italy) 10 mg once daily that was taken before dinner for 26 weeks and adherence to treatment was assessed by pill counts of pill containers that were provided to the patients. The dose of monacolin K was chosen according to that used in most studies conducted in patients with hypercholesterolemia [15]. All patients gave their written informed consent to the study. The study was conducted according to the principles of the Declaration of Helsinki and received approval from the local Institutional Review Board.

### 2.2. Laboratory Measurements

At baseline and at end of follow-up, blood was collected by venipuncture into silicone-treated glass tubes after an overnight fast. After separation, plasma was stored at −80 °C until assay. Measurements were taken as previously described [16]. Briefly, total cholesterol and triglycerides were assayed enzymatically by an automated method (International Laboratory, Milan, Italy). HDL-cholesterol was assayed after magnesium chloride-dextran sulphate precipitation of apolipoprotein B containing lipoproteins. The concentration of LDL-cholesterol was calculated with the formula of Friedewald et al. Glomerular filtration rate was assessed by duplicate measurement of 24 h creatinine clearance and normalized for body surface area [17]. Plasma glucose was assayed using the glucose oxidase method and plasma insulin was measured by radioimmunoassay. The homeostatic model assessment (HOMA) index was calculated as an index of insulin sensitivity from fasting plasma glucose (mmol/L) and insulin (μU/mL) using the formula: [(glucose × insulin)/22.5)] [18]. Plasma malondialdehyde and plasma-oxidized glutathione were measured by use of colorimetric assays (respectively: malondialdehyde microplate assay kit, Oxis Research OX 21034; and glutathione assay kit, Oxis Research OX21039; Portland, OR, USA) as markers of lipid peroxidation and oxidative stress [19].

### 2.3. Liver Steatosis Scores and Liver Ultrasound Elastography

Liver steatosis was assessed by 2 different biochemical scores: the fatty liver index (FLI) and hepatic steatosis index (HSI) (Table 1) [20]. These scores were validated in population studies [21,22].

Liver stiffness was assessed by acoustic radiation force impulse (ARFI) elastography using a standard machine (Siemens Acuson S2000TM ultrasound system; Siemens AG, Munich, Germany) and a 4 CI ultrasound probe. ARFI was performed twice in fasted patients by an expert operator (G.S.) that was blind to the patients’ characteristics [23]. Real-time B-mode imaging was used to ensure accuracy of sampling. Scanning was performed in the right liver lobe, with patients in supine position, 1–2 cm under the liver capsule, with minimal scanning pressure applied by the operator. All ARFI measurements were obtained at the end of deep inspiration. In all patients, 20 valid acquisitions for each site were obtained and the average value was considered. Measurement are expressed as m/s.

### 2.4. Bioimpedance Analysis

BIA was conducted using a standardized procedure by the same experienced operator (A.D.P.) that was blind to the patients’ characteristics. A fixed frequency device SECA (model mBCA 525; Seca gmbh & Co., Hamburg, Germany) and a tetrapolar method was used, as previously described [24]. Height and weight were measured on admission using a digital balance with altimeter, with patients wearing light clothes. Examination was carried out in patient lying supine, with legs, hands and arms away from the body. Four adhesive and disposable electrodes were placed on the dorsal surface of the hand and right foot on dry and disinfected skin at predetermined anatomical sites. The resistance (R), reactance (Xc) and phase angle (PA) were measured.

### 2.5. Statistical Analysis

Normality of distribution of the study variables was verified by the Kolmogorov–Smirnov test. Normally distributed variables are expressed as mean ± standard deviation and variables with skewed distribution as median [interquasrtile range, IQR]. Comparison of variables that were measured at baseline and at the end of follow-up was carried out by the Student’s *t* test for paired data for normally distributed variables and the Wilcoxon sum rank test for variables with skewed distribution. Patients with missing data were excluded from analysis. Changes were considered significant with a two-tailed probability of less that 0.05. Data analyses were performed using XL-STAT 2020 (Addinsoft, Paris, France).

## 3. Results

### 3.1. Characteristics of Patients

Thirty patients with NAFLD were evaluated for inclusion and six were excluded for missing laboratory values or follow-up drop-outs. We, therefore, included in analysis data of 24 patients, 10 males (42%) and 14 females (58%) aged 51 ± 12 years. Obesity (BMI ≥ 30) was present in 10 (42%) of the study participants and overweight (BMI ≥ 26) in all the remaining 14 (58%). History of hypertension was present in 12 (50%) of patients who were treated with renin-angiotensin system blockers (*n* = 8), calcium-channel blockers (*n* = 5), diuretics (*n* = 3) and beta-blockers (*n* = 3). Additional features suggestive of insulin resistance were present in 11 (46%) patients with an HOMA-index > 2.5. A baseline analysis of body composition revealed an elevated fat mass index with an elevated amount of visceral adipose tissue in all patients. ARFI elastography indicated increased liver stiffening in all patients with an average baseline value of 1.84 ± 0.93.

### 3.2. Outcomes of Monacolin K Treatment

Changes in clinical and biochemical variables of NAFLD patients that were observed after 26 weeks of treatment with monacolin K are summarized in Table 2. Neither BMI nor waist circumference had significant changes and also, systolic and diastolic blood pressure were comparable at baseline and end of follow-up. Fasting glucose and insulin levels remained unchanged, while the HOMA-index had a mild but significant decrease. Plasma triglycerides, total cholesterol and LDL-cholesterol were all significantly reduced by monacolin K administration, and among liver tests, only the ALT was significantly decreased.

Table 3 summarizes the data of BIA, showing that no significant changes of fat mass, visceral adipose tissue, skeletal muscle mass, and extracellular and total body water were observed with monacolin K treatment. Assessment of steatosis scores showed a significant improvement of the FLI, whereas the HSI had a mild and nonsignificant decrease (Table 4). Additionally, ARFI elastography showed a trend to improve (by 20%), but due to a wide dispersion of data, change was not significant (Table 4).

**Figure 1:**
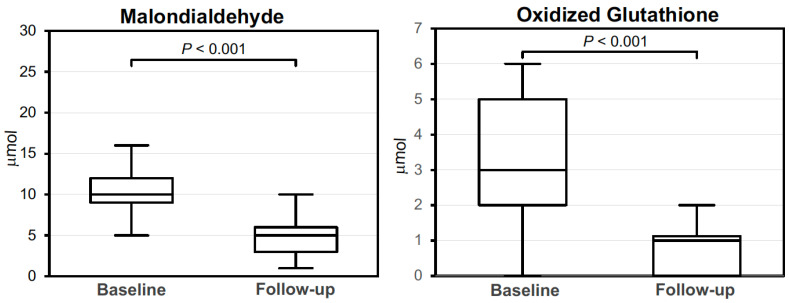
Box plot of baseline and follow-up plasma malondialdheyde and oxidized glutathione levels in 24 NAFLD patients that were treated for 26 weeks with monacolin K.

As shown in Figure 1, plasma levels of malondialdehyde and oxidized glutathione were markedly and significantly decreased at the end of follow-up.

## 4. Discussion

In this prospective, observational study, we tested the effects of monacolin K on plasma lipids, body composition and plasma markers of oxidative stress in a selected group of nondiabetic patients with NAFLD and mild hypercholesterolemia. Our findings demonstrate that use of 10 mg/day of monacolin K for 26 weeks significantly improved the plasma lipid profile, insulin sensitivity and biochemical steatosis scores in these patients, but it did not affect the body composition in terms of fat mass and visceral adipose tissue, nor liver elastographic characteristics. Plasma levels of malondialdehyde and oxidized glutathione were significantly reduced by monacolin K, suggesting a significant beneficial effect of this molecule on control of oxidative stress.

Over the past three decades, NAFLD became the most common chronic liver condition and is recognized to have a close relationship with most components of the metabolic syndrome [25]. NAFLD is frequently associated with obesity (51%), type-2 diabetes (22%), hypertension (39%) and hyperlipidemia (69%) [26]. Although no more than 10% of patients with NAFLD progress to more severe liver disease, due to its high prevalence in the adult population, NAFLD is now the most frequent cause of end-stage liver disease requiring liver transplantation with an ever increasing economic burden [25]. Liver biopsy is the gold standard for correct assessment of the extent of liver fat accumulation and permits identification of inflammatory changes and fibrosis that anticipate progression to more severe stages of disease. However, in consideration of the high frequency of NAFLD, systematic access to liver biopsy is unsuitable because of invasiveness, poor acceptability and costs. This is why, in recent years, alternative noninvasive strategies were proposed, including a physical approach with assessment of liver stiffness by ultrasound elastography and a biochemical approach based upon measurement of plasma biomarkers in association with anthropometric variables [27]. Because these noninvasive tools are widely utilized in clinical practice, liver steatosis was detected in our patients by use of two different biochemical scores (FLI and HSI) and ARFI elastography. These measurements were repeated after 26 weeks of use of monacolin K reporting a significant reduction in FLI, while HIS and ARFI had only a nonsignificant trend to improvement.

Currently, there is no FDA- or European Medicines Agency (EMA)-approved pharmacologic treatment for NAFLD and, therefore, lifestyle changes, including dietary modifications with weight loss and regular exercise, remain the cornerstone for therapy. Nonetheless, a large number of patients fail to benefit from lifestyle interventions and this is why many drugs were used to test their capability to be beneficial in NAFLD/NASH patients [28]. Because insulin resistance is directly involved in excess hepatic fat accumulation, many types of insulin sensitizers were tested and histological benefits of thiazolidinediones were demonstrated in patients with NASH [29]. The same benefit was not reported with metformin, but other newer antidiabetic agents such as glucagon-like peptide-1 agonists and SGLT2 inhibitors are currently under the magnifying glass. Other therapeutic approaches involved antioxidants, and reduction in liver fat deposition and inflammatory changes were demonstrated with vitamin E supplementation in NASH [29]. Additionally, modulators of nuclear transcription factors, cytokines and lipotoxicity contrasting agents were tested with minor benefits [28]. In particular, among the latter agents, statins [30] and omega-3polyunsaturated fatty acids were reported to show only marginal improvement of steatosis [31].

Because some nutraceuticals could contribute to a reduction in hepatic fat accumulation, many of these compounds were tested in patients with NAFLD. Recently, effects of silymarin, astaxanthin, coenzyme Q10, berberine, curcumin and resveratrol were systematically reviewed, reporting possible benefits if appropriately dosed and associated with lifestyle modifications [11]. Among nutraceuticals, the extract of red yeast rice is very effective in cholesterol lowering, and its effect can be attributed to the presence of a relatively high content of monacolins [32]. Monacolins are a group of substances that are produced from cold-fermentation of red yeast rice and different subtypes can be obtained based on the strain of yeast used. Monacolin K is one of these subtypes that was found to be structurally similar to lovastatin and, despite important differences in pharmacokinetic profiles, shares with lovastatin important lipid-lowering effects. This is why we sought to test the effects of monacolin K in NAFLD and the improvement of plasma lipid patterns observed in this study was not unexpected; especially if we consider that monacolin K acts by competitively blocking the activity of HMG-CoA reductase, the rate limiting enzymatic step in the mevalonate pathway of cholesterol synthesis. Therefore, by reducing cholesterol content in hepatocytes and like most statins, monacolin K increases LDL-receptor synthesis and transfer to the cell surface, thereby increasing LDL remnants removal from the bloodstream [33]. Thus, the present findings are in full agreement with those of previous studies [15,34] and support the evidence that monacolin K is an effective molecule for hypercholesterolemia and a reasonable alternative for treatment of statin-intolerant patients. It is well known that current guidelines of international societies for treatment of hypercholesterolemia recommend use of statins as the first step [35]. This is supported by extensive evidence showing the benefits of statins in the prevention of major cardiovascular events. This same evidence is not available in equal measure for other molecules that effectively decrease blood cholesterol. However, intolerance or refusal of patients to take statins frequently leads to use of alternative treatments including other drugs or dietary supplements. In this context and as previously stated, monacolin K shares the same mechanism of action of statins and carries a similar risk of intolerance because of metabolic interference in the skeletal muscle.

As stated above, decreased insulin sensitivity in hepatocytes as in other metabolic target tissues for this hormone causes compensatory hyperinsulinemia and up-regulation of lipogenic genes in the liver, leading to increased intracellular deposit of free fatty acids and triglycerides [36]. Experimental studies conducted in a mice model of NAFLD demonstrated that chronic administration of monacolin K reduced circulating insulin levels, obesity-related inflammatory markers and liver fat deposits, thus slowing progression of NAFLD to NASH [13]. Other studies tested the effects of monacolin K in high-fat diet-induced obese mice, reporting suppression of adipogenic and inflammatory genes that attenuated pathological adipose tissue remodeling and hepatic fat accumulation [37]. In line with these animal data, treatment with monacolin K induced a significant decrease in the HOMA-index of our NAFLD patients, supporting the hypothesis of a possible benefit on insulin sensitivity. Additionally, the significant reduction in ALT levels that was observed with monacolin K treatment might be expression of attenuation of the inflammatory response that is triggered in the liver by lipid accumulation and causes inflammatory cell infiltration and cell apoptosis [38].

Among the mechanisms that could cause NAFLD and its progression to NASH and liver cirrhosis, oxidative stress is considered to give a substantial contribution [39,40,41]. An imbalance between production of reactive oxygen and nitrogen species and antioxidant molecules was demonstrated in NAFLD [10]. This imbalance can cause hepatocellular damage by inhibition of the mitochondrial respiratory chain enzymes. Reactive oxygen species activate lipid peroxidation and local cytokine production and by triggering inflammatory responses and promoting fibrosis facilitate progression of disease. In our NAFLD patients, we measured plasma levels of malondialdehyde and oxidized glutathione as markers of oxidative stress and found that both were markedly decreased by monacolin K administration. Malondialdehyde is generated by peroxidation of cell membrane polyunsaturated fatty acids and was traditionally used as a tracer of lipid peroxidation. Increased serum malondialdehyde was reported in 67 patients with NAFLD or NASH in comparison to healthy controls [39] and in a study of 32 patients with biopsy-proven NAFLD, serum and hepatic malondialdehyde levels were significantly associated with the HOMA-index and risk of progression to NASH [40]. Glutathione is one of the most powerful antioxidant molecules and its depletion was reported in patients with NAFLD [42]. In a study conducted in Japan, genetic variants of the glutathione-S-transferase, an enzyme that is crucially important for antioxidant defense mechanisms, were associated with the development of NAFLD [43]. Moreover, protein glutathionylation occurring in hepatocytes in response to oxidative stress was significantly increased in livers with NAFLD [44], strongly supporting the view that oxidative injury plays a crucial role in this condition.

Although the findings of this study strongly suggest that monacolin K could have some modulatory effects on oxidative stress, no clear evidence of benefits on liver structure could be found. The improvement of one of the biochemical scores of liver steatosis (FLI) could be easily explained by a decrease in plasma triglyceride levels and both liver elastography and BIA did not detect any significant changes with use of monacolin K. Although the duration of the study might have been too short to detect significant changes, further studies will be needed to better explore the possibility that monacolin K could be beneficial in NAFLD.

Important limitations of the study must be acknowledged including the weakness of the open design without a placebo control group, together with the relatively small size. It has to be considered that this study was conceived as a pilot exploratory experience with the aim of generating hypotheses that could be further explored in appropriately structured investigations. Additionally, the duration of the study might have limited the possibility to detect significant changes in liver elastographic characteristics and body fat mass. The lack of control for dietary factors during the study period might have affected some of the results. However, as detailed above, during the study, the NAFLD patients received the same dietary recommendations that they were receiving for a long time (at least one year) before inclusion in the study. One more point is related to limitations that biochemical scores of liver steatosis and liver elastography carry in comparison to liver biopsies for definition of the extent of steatosis. Although biochemical scores and elastography are widely employed, liver histology is the gold standard that permits precise assessment of degree of steatosis together with detection of inflammatory changes and fibrosis as markers of possible evolution of NAFLD to more severe stages of liver disease [27]. Additionally, the availability of tissue samples would permit direct assessment of markers of oxidative stress that would provide better information with regard to its involvement in NAFLD and to potential benefits of treatment. Lastly, we did not measure plasma levels of free fatty acids and adipocytokines that would have been useful to provide further interpretation of our present findings.

## 5. Conclusions

In this prospective, observational, open study, we tested the effects of monacolin K in patients with NAFLD. The findings indicated that administration of monacolin K for 26 weeks reduced plasma lipids but did not significantly affect the body fat mass and visceral adipose tissue, nor liver elastographic characteristics. Nonetheless, the reduction in the FLI and improvement of insulin sensitivity indicated a possible reduction in liver fat deposition. Plasma malondialdehyde and oxidized glutathione were significantly reduced by monacolin K, suggesting a significant benefit of the reduction in oxidative stress and lipid peroxidation. This pilot study suggested possible benefits of monacolin K use in NAFLD patients that could be linked to improvement of insulin sensitivity and reduction in oxidative stress. This initial evidence will need further assessment in appropriately designed studies.

## Figures and Tables

**Table 1:** Biochemical scores that were used for definition of liver steatosis.

FLI	Body mass index (BMI) Waist circumference, cm Serum triglycerides, mg/dL Serum gamma-glutamyl transpeptidase (GGT), U/L	(e^0.953∗log^_e_^(triglycerides)+0.139∗BMI+0.718∗log^_e_^(GGT)+0.053∗waist circumference−15.745^)/(1 + e^0.953∗log^_e_^(triglycerides)+0.139∗BMI+0.718∗log^_e_^(GGT)+0.053∗waist circumference−15.745^) × 100	<30 steatosis excluded >60 indicates steatosis
HSI	GenderBody mass index (BMI) Aspartate transaminase (AST), U/L Alanine transaminase (ALT), U/L Type 2 diabetes	8 × ALT/AST + BMI (+2 if type 2 diabetes present, +2 if female)	<30 steatosis excluded >36 indicates steatosis

FLI, fatty liver index; HSI, hepatic steatosis index; BMI, body mass index; GGT, gamma-glutamyl transpeptidase; AST, aspartate aminotransferase; ALT, alanine aminotransferase.

**Table 2:** Clinical and biochemical variables of NAFLD patients that were treated for 26 weeks with monacolin K at baseline and at end of follow-up.

Variable	Baseline	Follow-Up	*p*
Body mass index, kg/m^2^	28.8 ± 4.0	27.7 ± 4.4	0.532
Waist circumference, cm	100.8 ± 7.8	100.1 ± 8.2	0.532
Systolic blood pressure, mm Hg	132.1 ± 10.5	132.7 ± 6.6	0.761
Diastolic blood pressure, mm Hg	81.7 ± 10.3	81.5 ± 6.8	0.794
Glucose, mg/dL	94.3 ± 12.4	93.8 ± 11.2	0.861
Insulin, mU/L	12.2 ± 7.0	11.3 ± 9.0	0.619
HOMA-index	2.9 ± 1.9	2.3 ± 1.1	0.036
Triglycerides, mg/dL	135 ± 99	130 ± 46	0.026
Cholesterol, mg/dL	232 ± 22	204 ± 35	0.003
HDL-cholesterol, mg/dL	52 ± 15	53 ± 11	0.727
LDL-cholesterol, mg/dL	144 ± 31	127 ± 29	0.012
AST, U/L	38 ± 25	29 ± 12	0.135
ALT, U/L	51 ± 26	34 ± 17	0.018
GGT, U/L	77 ± 36	58 ± 57	0.164
AP, U/L	80 ± 35	80 ± 33	0.662
Bilirubin, mg/dL	0.65 ± 0.25	0.61 ± 0.28	0.597
Albumin, g/dL	43.3 ± 4.6	44.7 ± 4.2	0.285

HOMA, homeostasis model assessment; HDL, high-density lipoprotein; LDL, low-density lipoprotein; AST, aspartate aminotransferase; ALT, alanine aminotransferase; GGT, gamma-glutamyl transpeptidase; AP, alkaline phosphatase.

**Table 3:** Bioimpedance analysis variables of NAFLD patients that were treated for 26 weeks with monacolin K at baseline and at end of follow-up.

Variable	Baseline	Follow-Up	*p*
Fat mass, kg	28.2 ± 10.4	26.3 ± 11.7	0.626
Fat mass, %	36.2 ± 10.9	31.8 ± 12.4	0.387
Fat mass index, kg/m^2^	10.2 ± 4.4	9.6 ± 4.8	0.525
Fat free mass, kg	53.2 ± 9.3	54.9 ± 9.7	0.579
Fat free mass, %	65.8 ± 10.9	68.2 ± 12.3	0.387
Fat free mass index, kg/m^2^	18.6 ± 1.6	19.2 ± 1.7	0.234
Skeletal muscle mass, kg	25.6 ± 5.7	26.4 ± 5.6	0.705
Total body water, L	39.5 ± 6.5	40.8 ± 6.7	0.561
Extracellular body water, L	17.2 ± 2.1	17.8 ± 2.2	0.350
Extracellular/total body water, %	43.8 ± 3.0	44.0 ± 3.1	0.989
Visceral adipose tissue, L	3.0 ± 0.8	3.0 ± 1.1	0.836
Phase angle, °	6.4 ± 0.9	6.4 ± 1.0	0.888

**Table 4:** Liver steatosis scores, liver stiffness, and markers of oxidative stress of NAFLD patients that were treated for 26 weeks with monacolin K at baseline and at end of follow-up.

Variable	Baseline	Follow-Up	*p*
FLI	75.7 ± 18.0	64.9 ± 20.4	0.035
HSI	40.6 ± 5.3	38.9 ± 4.9	0.317
ARFI	1.84 ± 0.93	1.48 ± 0.99	0.234

FLI, fatty liver index; HIS, hepatic steatosis index; ARFI, acoustic radiation force impulse.

## Data Availability

Not applicable.

## References

[1] Marchesini G., Day C.P., Dufour J.F., Canbay A., Nobili V., Ratziu V., Tilg H., Roden M., Gastaldelli A., Yki-Jarcvienen H. (2016). EASL-EASD-EASO Clinical Practice Guidelines for the management of non-alcoholic fatty liver disease. J. Hepatol..

[2] Vancells Lujan P., Vinas Esmel E., Sacanella Meseguer E. (2021). Overview of non-alcoholic fatty liver disease (NAFLD) and the role of sugary food composition and other dietary components in its development. Nutrients.

[3] Vernon G., Baranova A., Younossi Z.M. (2011). Systematic review: The epidemiology and natural history of non-alcoholic fatty liver disease and non-alcoholic steatohepatitis in adults. Aliment. Pharmacol. Ther..

[4] Godoy-Matos A.F., Silva W.S., Valerio C.M. (2020). NAFLD as a continuum: From obesity to metabolic syndrome and diabetes. Diabetol. Metab. Syndr..

[5] Eslam M., El-Serag H.B., Francque S., Sarin S.K., Wei L., Bugianesi E., George J. (2022). Metabolic (dysfunction) associated fatty liver disease in individuals of normal weight. Nat. Rev. Gastroenterol. Hepatol..

[6] Yki-Järvinen H. (2014). Non-alcoholic fatty liver disease as a cause and a consequence of metabolic syndrome. Lancet Diabetes Endocrinol..

[7] Bellanti F., Villani R., Facciorusso A., Vendemiale G., Serviddio G. (2017). Lipid oxidation products in the pathogenesis of non-alcoholic steatohepatitis. Free Radic. Biol. Med..

[8] Polimeni L., Del Ben M., Baratta F., Perri L., Albanese F., Pastori D., Violi F., Angelico F. (2015). Oxidative Stress: New insights on the association of non-alcoholic fatty liver disease and atherosclerosis. World J. Hepatol..

[9] Mansouri A., Gattolliat C.-H., Asselah T. (2018). Mitochandrial dysfunction and signalling in chronic liver disease. Gastroenterology.

[10] Yang J., Fernandez-Galilea M., Martinez-Fernandez L., Gonzalez-Muniesa P., Perez-Chavez A., Martinez J.A., Moreno-Aliaga M.J. (2019). Oxidative stress and non-alcoholic fatty liver disease: Effects of omega-3 fatty acid supplementation. Nutrients.

[11] Cicero A.F.G., Colletti A., Bellentani S. (2018). Nutraceutical approach to non-alcoholic fatty liver disease (NAFLD): Tha available clinical evidence. Nutrients.

[12] Xiong Z., Cao X., Wen Q., Chen Z., Cheng Z., Huang X., Zhang Y., Long C., Zhang Y., Huang Z. (2019). An overview of monacolin K/lovastatin. Food Chem. Toxicol..

[13] Fujimoto M., Tsuneyama K., Chen S.-Y., Nishids T., Chen J.-L., Chedn Y.-C., Fujimoto T., Imura J., Shimada Y. (2012). Study of the effects of monacolin K and other constituents of red yeast rice on obesity, insulin-resistance, hyperlipidemia, and nonalcoholic steatohepatitis using a mouse model of metabolic syndrome. Evid. Based Complement. Alternat. Med..

[14] Parra-Vargas M., Rodriguez-Ecchevarria R., Jimenez-Chillaron J.C. (2020). Nutritional approaches for the management of nonalcoholic fatty liver disease: And evidence-based review. Nutrients.

[15] Stefanutti C., Mazza F., Mesce D., Morozzi C., Di Giacomo S., Vitale M., Pergolini M. (2017). Monascus purpureus for statin and ezetimibe intolerant heterozygous familial hypercholesterolemia patients: A clinical study. Atheroscler. Suppl..

[16] Brosolo G., Catena C., Da Porto A., Bulfone L., Vacca A., Verheyen N.D., Sechi L.A. (2022). Differences in regulation of cortisol secretion contribute to left ventricular abnormalities in patients with essential hypertension. Hypertension.

[17] Catena C., Colussi G.L., Brosolo G., Verheyen N.D., Novello M., Bertin N., Cavarape A., Sechi L.A. (2017). Long-term renal and cardiac outcomes after stenting in patients with resistant hypertension and atherosclerotic renal artery stenosis. Kidney Blood Press. Res..

[18] Brosolo G., Da Porto A., Bulfone L., Scandolin L., Vacca A., Bertin N., Vivarelli C., Sechi L.A., Catena C. (2022). Vitamin D deficiency is associated with glycometabolic changes in nondiabetic patients with arterial hypertension. Nutrients.

[19] Soardo G., Donnini D., Varutti R., Moretti M., Milocco C., Basan L., Esposito W., Casaccio D., Stel G., Catena C. (2005). Alcohol-induced endothelial changes are associated with oxidative stress and are rapidly reversed after withdrawal. Alcohol. Clin. Exp. Res..

[20] Catena C., Brosolo G., Da Porto A., Donnini D., Bulfone L., Vacca A., Soardo G., Sechi L.A. (2022). Association of non-alcoholic fatty liver disease with left ventricular changes in treatment-naïve patients with uncomplicated hypertension. Front. Cardiovasc. Med..

[21] Fedchuck L., Nascimbeni F., Pais R., Charlotte F., Housset C., Ratziu V. (2014). LIDO Study Group. Performance and limitations of steatosis biomarkers in patients with nonalcoholic fatty liver disease. Aliment. Pharmacol. Ther..

[22] Younes R., Caviglia G.P., Govaere O., Rosso C., Armandi A., Sanavia T., Pennisi G., Liguori A., Francione P., Gallego-Durán R. (2021). Long-term outcomes and predictive ability of noninvasive scoring systems in patients with nonalcoholic fatty liver disease. J. Hepatol..

[23] Sagir A., Ney D., Oh J., Pandey S., Kircheis G., Mayatepek E., Haussinger D. (2015). Evaluation of acoustic radiation force impulse imaging (ARFI) for the determination of liver stiffness using transient elastography as a reference in children. Ultrasound Int. Open.

[24] Da Porto A., Tascini C., Peghin M., Sozio E., Colussi G.L., Casarsa V., Bulfone L., Graziano E., De Carlo E., Catena C. (2021). Prognostic role of malnutrition diagnosed by bioelectrical impedance vector analysis in older adults hospitalized with COVID-19 pneumonia: A prospective study. Nutrients.

[25] Powell E.E., Wong V.W.-S., Rinella M. (2021). Non-alcoholic fatty liver disease. Lancet.

[26] Younossi Z.M., Koenig A.B., Abdelatif D., Fazel Y., Henry L., Wymer M. (2016). Global epidemiology of nonalcoholic fatty liver disease-meta-analytic assessment of prevalence, incidence, and outcomes. Hepatology.

[27] Castera L., Friedrich-Rust M., Loomba R. (2019). Noninvasive assessment of liver disease in patients with nonalcoholic fatty liver disease. Gastroenterology.

[28] Raza S., Rajak S., Upadhyay A., Tewari A., Sinha R.A. (2021). Current treatment paradigms and emerging therapies for NAFLD/NASH. Front. Biosci..

[29] Sanyal A.J., Chalasani N., Kowdley K.V., McCullough A., Diehl A.M., Bass N.M., Neuschwander-Tetri B.A., Lavine J.E., Tonascia J., Unalp A. (2010). Pioglitazone, vitamin E, or placebo for nonalcoholic steatohepatitis. N. Engl. J. Med..

[30] Ekstedt M., Franzen L.E., Mathiasen U.L., Holmqvisat M., Bodemar G., Kechagias S. (2007). Statins in non-alcoholic fatty liver disease and chronically elevated liver enzymes: A histopathological follow-up study. J. Hepatol..

[31] Chen L.H., Wang Y.F., Xu Q.H., Chen S.S. (2018). Omega-3 fatty acids as a treatment for non-alcoholic fatty liver disease iin children. Clin. Nutr..

[32] Cicero A.F.G., Fogacci F., Banach M. (2019). Red yeast rice for hypercholesterolemia. Methodist Debakey Cardiovasc. J..

[33] Poli A., Barbagallo C.M., Cicero A.F.G., Corsini A., Manzato E., Trimarco B., Bernini F., Visioli F., Bianchi A., Canzone G. (2018). Nutraceuticals and functional foods for the control of plasma cholesterol levels. An intersociety position paper. Pharmacol. Res..

[34] Gerards M.C., Terlou R.J., Yu H., Koks C.H.W., Gerdes V.E.A. (2015). Traditional Chinese lipid-lowering agent red yeast rice results in significant LDL reduction but safety is uncertain. A systematic review and meta-analysis. Atherosclerosis.

[35] Grundy S.M., Stone N.J., Bailey A.L., Beam C., Birtcher K.K., Blumenthal R.S., Braun L.T., de Ferranti S., Faiella-Tommasino J., Forman D.E. (2019). 2018 AHA/ACC/AACVPR/AAPA/ABC/ACPM/ADA/AGS/APhA/ASPC/NLA/PCNA guideline on thje management of blood cholesterol. Circulation.

[36] Pierantonelli I., Svegliati-Baroni G. (2019). Nonalcoholic fatty liver disease: Basic pathogenetic mechanisms in the progression from NAFLD to NASH. Transplantation.

[37] Munkong N., Lonan P., Mueangchang W., Yadyookai N., Kanjoo V., Yoysungnoen B. (2022). Red rice bran extract attenuates adipogenesis and inflammation on white adipose tissues in high-fat diet-induced obese mice. Foods.

[38] Teng T., Qiu S., Zhao Y., Zhao S., Sun D., Hou L., Li Y., Zhou K., Yu X., Yang C. (2022). Pathogenesis and therapeutic strategies related to non-alcoholic fatty liver disease. Int. J. Mol. Sci..

[39] Świderska M., Maciejczyk M., Zalewska A., Pogorzelska J., Flisiak R., Chabowski A. (2019). Oxidative stress biomarkers in the serum and plasma of patients with non-alcoholic fatty liver disease (NAFLD). Can plasma AGE be a marker of NAFLD? Oxidative stress biomarkers in NAFLD patients. Free Radic. Res..

[40] Köroǧlu E., Canbakan B., Atay K., Hetemi I., Tuncer M., Dobrucali A., Sonsuz A., Gultepe I., Senturk H. (2016). Role of oxidative stress and insulin resistance in disease severity of non-alcoholic fatty liver disease. Turk. J. Gastroenterol..

[41] Soardo G., Donnini D., Domenis L., Catena C., De Sivestri D., Cappello D., Dibenedetto A., Carnelutti A., Bonasia V., Pagano C. (2011). Oxidative stress is activated by free fatty acids in cultured human hepatocytes. Metab. Syndr. Relat. Disord..

[42] Lieber C.S., Leo M.A., Mak K.M., Xu Y., Cao Q., Ren C., Ponomarenko A., De Carli L.M. (2004). Model of nonalcoholic steatohepatitis. Am. J. Clin. Nutr..

[43] Hori M., Oniki K., Nakagawa T., Takata K., Mihara S., Marubayashi T., Nakagawa K. (2009). Association between combinations of glutathione-S-transferase M1, T1 and P1 genotypes and non-alcoholic fatty liver disease. Liver Int..

[44] Piemonte F., Petrini S., Gaeta L.M., Tozzi G., Bertini E., Devito R., Boldrini R., Marcellini M., Ciacco E., Nobili V. (2008). Protein glutathionylation increases in the liver of patients with non-alcoholic fatty liver disease. J. Gastroenterol. Hepatol..

